# 200 years of taxonomic confusion:* Sporendonema* and allies

**DOI:** 10.1007/s10482-024-01935-3

**Published:** 2024-03-14

**Authors:** Hazal Kandemir, Cony Decock, Margarita Hernández-Restrepo, Roman Labuda, Jos Houbraken, Macit Ilkit, G. Sybren de Hoog

**Affiliations:** 1 Center of Expertise in Mycology, Radboud University Medical Center/Canisius Wilhelmina Hospital, Nijmegen, The Netherlands; 2 Westerdijk Fungal Biodiversity Institute, Utrecht, The Netherlands; 3 Mycothéque de l’Université Catholique de Louvain, Earth and Life Institute, Louvain-La-Neuve, Belgium; 4 Research Platform Bioactive Microbial Metabolites, Tulln/Donau, Austria; 5 Institute of Food Safety, Food Technology and Veterinary Public Health, University of Veterinary Medicine Vienna, Veterinaerplatz 1, 1210 Vienna, Austria; 6 Division of Medical Mycology, Faculty of Medicine, University of Çukurova, Adana, Turkey

**Keywords:** *Arachniotus*, Cheese fungi, Halotolerance, *Onygenales*, *Sphaerosporium*, Taxonomy

## Abstract

The genus *Sporendonema* (*Gymnoascaceae*, *Onygenales*) was introduced in 1827 with the type species *S*. *casei* for a red mould on cheese. Cheese is a consistent niche for this species. *Sphaerosporium equinum* is another species classified in *Gymnoascaceae* and has also been reported from cheese. Recently, other habitats have been reported for both *Sporendonema casei* and *Sphaerosporium equinum*. The present study aimed to investigate the taxonomy of *Sporendonema* and *Sphaerosporium*, as well as a close neighbour, *Arachniotus*. Two strains of *Hormiscium aurantiacum*, another related cheese-associated species were also included in the analyses. Strains were evaluated in terms of macro- and micromorphology, physiology including salt tolerance, growth rate at different temperatures, casein degradation, cellulase activity, lipolytic activity, and multi-locus phylogeny with sequences of the nuclear ribosomal internal transcribed spacer region, the D1-D2 region of the large subunit and partial β-tubulin locus sequences. The results showed that the analysed species were congeneric, and the generic names *Arachniotus* and *Sphaerosporium* should be reduced to the synonymy of *Sporendonema*. Therefore, four new combinations as well as one lectotype and one epitype were designated in *Sporendonema*. Two strains attributed to *Sphaerosporium equinum* from substrates other than cheese were found to be phylogenetically and morphologically deviant and were introduced as a new species named *Sporendonema isthmoides*.

**Supplementary Information:**

The online version contains supplementary material available at 10.1007/s10482-024-01935-3.

## Introduction

Molecular systematics and comparative genomics in mycology provide tools to reevaluate large groups of fungi that were previously described on the basis of morphology alone, or with limited molecular data. Additionally, orders that contain medically important species, such as *Mucorales* (Chibucos et al. 2016; Walther et al. 2019), *Chaetothyriales* (Quan et al. 2020), *Hypocreales* (Kepler et al. 2017; Crous et al. 2021), and *Onygenales* (Kandemir et al. 2022), have been studied by applying a multiphasic approach combining multi-locus phylogenies, morphology, physiology, and ecology of the species. Results of experimental studies tend to lead to numerous taxonomic and nomenclatural changes. These rearrangements inevitably forced researchers to redefine generic and specific concepts, and to revise the criteria for new species (Aime et al. 2021). The use of molecular (e.g., barcoding genes) and morphological (e.g., culture characteristics and sexual and asexual morphs) data to delimit species, genera and families, as addressed in several studies, ultimately should clarify and stabilize nomenclature (Zamora et al. 2018; Kandemir et al. 2020; Lücking et al. 2020; Jiang et al. 2020; Crous et al. 2021).

In a revision of the order *Onygenales*, Kandemir et al. (2022) provided an overview of the ecological, morphological and phylogenetic characteristics of its families. Several pending nomenclatural and taxonomical issues have been solved in recent papers (Hainsworth et al. 2021; Labuda et al. 2021; Rodríguez-Andrade et al. 2021). However, some issues remain unsolved, among which is *Sporendonema* and relatives in the family *Gymnoascaceae*. Molecular data shows that, besides *Sporendonema* (*S*.) *casei*, the genus includes species previously classified in *Arachniotus* and *Sphaerosporium* (Kandemir et al. 2022). *Sphaerosporium* (*Sph*.) *equinum* and *S*. *casei* were isolated mainly from cheese and dried meat products and are known to be halophilic (Ropars et al. 2012; Scaramuzza et al. 2015), whereas members of *Arachniotus* (*A*.) are mainly isolated from dung and agricultural soil. However, *A*. *desertorum* was originally isolated from halomorphic soil in Kuwait (Moustafa 1973). In the present study, we aimed to investigate the relationship among the genera *Arachniotus*, *Sphaerosporium*, and *Sporendonema* using nuclear ribosomal internal transcribed spacer (ITS), D1-D2 region of the large subunit (LSU), and partial β-tubulin (*TUB*) phylogenies, in addition to morphological and physiological data.

## Materials and methods

### Strains

Strains were obtained from the CBS collection (housed at the Westerdijk Fungal Biodiversity Institute, Utrecht, The Netherlands), the Mycothèque de l’Université Catholique de Louvain (BCCM/MUCL; Louvain-la-Neuve, Belgium), and the University of Alberta Microfungus Collection and Herbarium (UAMH; currently in Toronto, Canada). Additionally, two specimens, ILLS 36355, isotype of *Hormiscium* (*H*.) *aurantiacum*, and ILLS 45141, isoneotype of *Torula* (*T*.) *equina*, were loaned from The Illinois Natural History Survey Herbarium (USA). Strain information is provided in Table 1.

**Table 1:** Sources and GenBank accession numbers of the strains used in the study

Current taxon name^1^	New taxon name	Collection number^2^	Source, country	GenBank accession numbers^3^		
				ITS	LSU	*TUB*
*A. aurantiacus*	*S*. *aurantiacum*	CBS 603.67^ T^	Soil, USSR	HM991267	AY176747	**ON075078**
		CBS 405.84	Dung of mouse, Netherlands	**OM468610**	**OM515116**	–
*A. confluens*	*S*. *confluens*	CBS 352.66^ T^	Dung, UK	AJ315837	**OM515113**	–
		CBS 634.72	Soil, Kuwait	MH860605	JQ434634	JQ434517
*A. ruber*	*S*. *rubrum*	CBS 352.90^ T^	Soil, UK	MH862216	MH873901	**OM047602**
		CBS 112.69	Wheat field soil, Germany	CBS database	MH878440	**OM616024**
		CBS 351.66	Alluvial pasture soil, UK	**OM468608**	**OM515114**	**OM616025**
		CBS 592.71	Agricultural soil, Netherlands	MH860278	MH872036	**OM616027**
*G. afilamentosa*	*G. afilamentosa*	CBS 658.71^ T^	Clay soil, USA	NR_160135	NG_057626	–
*G. anodosa*	*G. anodosa*	CBS 518.68^ T^	Rabbit dung, USA	MH859182	–	–
		CBS 517.68	Fox dung, USA	MH859181	MH870900	–
*G. aurantiaca*	*G. aurantiaca*	UAMH 3138^ T^	Lizard dung, Mexico	AJ315834	–	–
		CBS 655.71	Clay soil, USA	NR_145221	AB040684	**ON075079**
*G. dankaliensis*	*G. dankaliensis*	CBS 117.38^ T^	Camel skin lesion, Italy	MH855927	MH867428	-
*G. littoralis*	*G. littoralis*	CBS 454.73^ T^	Conch shell, Canada	MH860738	MH872451	–
*G. nodulosa*	*G. nodulosa*	CBS 577.63^ T^	Guinea pig dung, India	NR_160094	NG_06403	–
*Gymnascella* sp.	*Gymnascella* sp.	CCF 6605	Dust sample, Austria	OL527727	OL527728	–
*G*. *stercoraria*	*G*. *stercoraria*	LC4076^T^	Compost, China	KP278214	KP278223	–
*G*. *thermotolerans*	*G*. *thermotolerans*	LC3877^T^	Corn field soil, China	KP278212	KP278221	–
*G. udagawae*	*G. udagawae*	CBS 950.69^ T^	Soil, Japan	MH859492	NG_064075	–
*Gym. petalosporus*	*Gym. petalosporus*	UAMH 1665^ T^	Human skin lesion, India	HM991270	AY176748	–
*Gymn. dugwayensis*	*Gymn. dugwayensis*	ATCC 18899^ T^	Sandy soil, USA	LC146737	–	–
*Gymn. exasperatus*	*Gymn. exasperatus*	LC5640^T^	Bat guano, China	KU746682	KU746728	KU746773
*Gymn. longitrichus*	*Gymn. reessii*	CBS 366.64^ T^	Composite soil, USA	MH858457	MH870091	–
		CBS 392.64^ T^	Soil, USA	MH858463	MH870096	–
		CBS 410.72	Soil, USA	MH860507	MH872224	**OM047603**
		LCP 60.1696	Soil dune, Iran	JQ434569	JQ434633	JQ434516
*Gymn. uncinatus*	*Gymn. uncinatus*	CBS 408.72^ T^	Dung, USA	KT155648	MZ437795	–
*H. aurantiacum*	*S*. *casei*	CBS 111.18	Stockfish, unknown	**OM468605**	**OM515117**	**OM616029**
		CBS 206.35	Cheese rind, Netherlands	MH855648	MH867157	–
*N. armeniaca*	*N. armeniaca*	CBS 125.78^ T^	Kangaroo dung, India	AJ315827	–	–
*N. echinulata*	*N. echinulata*	IFO 9192 T	Unknown	AJ271562	–	–
*N. hyalinospora*	*N. hyalinospora*	CBS 548.72^ T^	Guinea pig dung, India	NR_130659	NG_057618	**OM047604**
*N. poonensis*	*N. poonensis*	CBS 393.71^ T^	Soil, India	MH860180	MH871950	–
*N. punctata*	*N. punctata*	CBS 279.64^ T^	Rice-field soil, India	AJ315825	AB075340	–
*Sph. equinum*	*S*. *equinum*	MUCL 31968	Cantal cheese, France	**OM468892**	**OM515120**	**ON075080**
		MUCL 38540	French cheese, France	**OM468893**	**OM515121**	**ON075085**
		MUCL 40624	Cheese, France	JQ434576	JQ434640	JQ434523
		MUCL 40625	Cheese, France	JQ434575	JQ434639	JQ434522
		MUCL 40795	Cheese, France	JQ434577	JQ434641	JQ434524
		MUCL 46080	Rind of sheep cheese, France	JQ434578	JQ434642	JQ434525
		MUCL 49171	Pyrenean cheese, Belgium	JQ434574	JQ434638	JQ434521
*S. casei*	*S. casei*	CBS 543.75^ET^	Cheese, unknown	MH860952	MH872720	**ON075084**
		CBS 143878	Sardinian Pecorino cheese, Italy	**OM468606**	**OM515122**	**ON075081**
		CBS 207.27	Unknown, France	MH854931	**OM515115**	**ON075082**
		CBS 355.29	Unknown	JQ434573	JQ434637	**ON075083**
		CBS 360.49	Cheese, unknown	**OM468609**	**OM515123**	**OM616028**
		HDN16-802	Sediment sample, China	MK578184	–	–
		isolate RS3	Cheese, Unknown	KF669522	–	–
		MUCL 38539	Cheese, France	JQ434572	JQ434636	JQ434519
***S. isthmoides***	***S. isthmoides***	MUCL 58097^ T^	Bat wing swab, Canada	**OM468607**	**OM515118**	**OM616026**
		MUCL 54024	Insect pupa, Belgium	**OK255531**	**OK255535**	–
*Art. ciferrii*	*Art. ciferrii*	CBS 272.66^ T^	Opossum hair and soil, USA	NR_144888	NG_057027	KT155525
*Art. crocatum*	*Art. crocatum*	IHEM 5251^ T^	Soil, Japan	LR136969	MZ645745	LR136785

^1^*A* = *Arachniotus*, *Art* = *Arthroderma*, *G* = *Gymnascella*, *Gym* = *Gymnoascoideus*, *Gymn* = *Gymnoascus*, *H* = *Hormiscium N* = *Narasimhella*, *Sph* = *Sphaerosporium*, *S* = *Sporendonema*, **bold** indicates new taxa. ^2^*CBS* Culture collection of the Westerdijk Biodiversity Institute, The Netherlands, *IFO* Institute for Fermentation Culture Collection, Japan, *IHEM* Mycology Laboratory of the Institute of Hygiene and Epidemiology, Belgium, *LCP* The Fungal Culture Collection of the Muséum National d'Histoire Naturelle, France, *MUCL* Mycothèque de l’Université Catholique de Louvain, Belgium, *NRRL* American Research Service culture collection, USA, *UAMH* University of Alberta Microfungus Collection and Herbarium, Canada. ^*ET*^ epitype strain. ^*T*^ type strain. ^3^*ITS* internal transcribed spacer nrDNA, *LSU* large subunit nrRNA, *TUB* partial beta-tubulin gene, sequences produced in this study are shown in **bold**

### DNA extraction, PCR and sequencing

The strains were grown for 14–21 d on malt extract agar (MEA) were used for DNA extraction using the Wizard® Genomic DNA purification Kit (Promega Corp., Madison, WI, USA), according to the manufacturer’s instructions. Three gene regions, ITS, LSU and *TUB*, were amplified using the primers ITS4-ITS5 (White et al. 1990, Ward and Adamas 1998), LR0R-LR5 (Vilgalys and Hester 1990) and TUB2Fd-TUB4Fd (Woudenberg et al. 2009), respectively. The PCR conditions were as follows: 35 cycles of 45 s at 94 °C, 45 s at 52 °C and 90 s at 72 °C for the ITS and LSU markers; 35 cycles of 45 s at 94 °C, 45 s at 48 °C and 90 s at 72 °C for the *TUB* region. PCR amplicons were visualized on 1.5% agarose gels. Sequencing was performed with the same primer pairs as used for PCR amplification using Applied Biosystems BigDye Terminator v.3.1 (Thermo Fisher Scientific).

### Molecular identification and phylogenetic analyses

Sequences for each marker were edited and assembled in Geneious R11 v.2022.0.1 (Kearse et al. 2012) and deposited in GenBank (Table 1). The sequences were aligned in MAFFT v.7 (Katoh et al. 2019) and combined with Sequence Matrix v.1.8 (Vaidya et al. 2011). In total, 54 isolates were included from which 54 ITS, 47 LSU, and 28 *TUB* sequences were analysed. The best-fitting model for each gene was found using ModelFinder (Kalyaanamoorthy et al. 2017) on the IQ-TREE web server (http://iqtree.cibiv.univie.ac.at/) (Nguyen et al. 2015) according to the Bayesian Information Criterion (BIC). Phylogenetic trees were constructed using MrBayes v.3.2.7 analyses on the CIPRES website (http://www.phylo.org) (Ronquist and Huelsenbeck 2003) and maximum likelihood (ML) methods implemented on the W-IQ-TREE web server (Trifinopoulos et al. 2016; Minh et al. 2020). All alignments and phylogenetic trees were deposited in TreeBASE (http://treebase.org; TB2:S29250) and figshare (10.6084/m9.figshare.23284661) repositories.

### Morphology

Morphology of the colonies was observed on potato dextrose agar (PDA) and yeast powder-soluble starch agar (YpSs) after 21 d of incubation at 24 °C in the dark. Since *S*. *casei* and *H*. *aurantiacum* strains were not able to grow at 24 °C, these strains were incubated at 15 °C in the dark for 21 days. Colony details and morphology of the fungarium specimens were observed using a Nikon SMZ1500 microscope, and microphotographs were taken by Nikon Eclipse80i equipped with a Nikon DSRi2 camera.

### Physiology

**Fig. 1:**
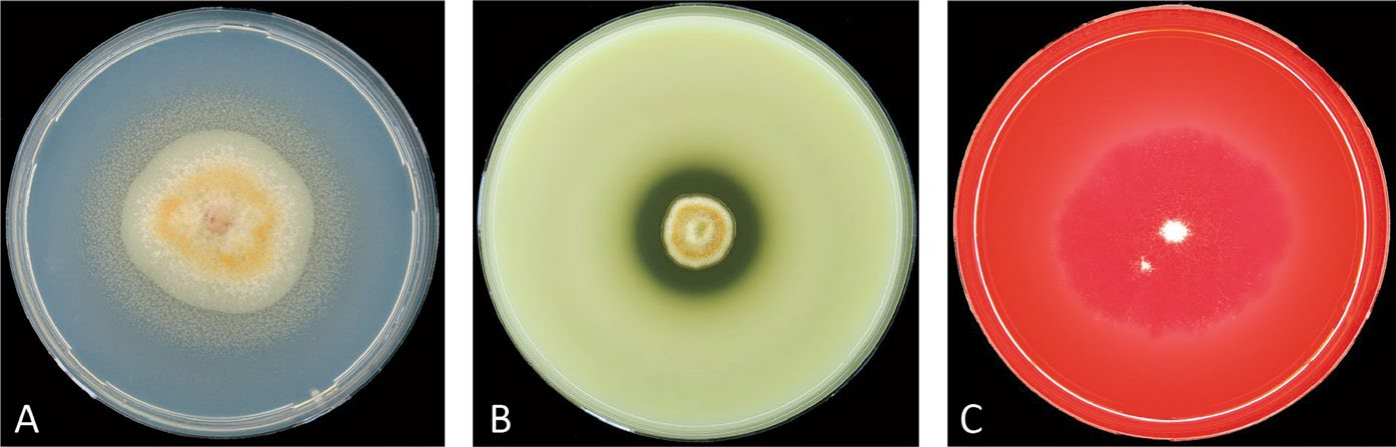
Interpretation of the lipolytic, proteolytic and cellulolytic activities. Positive results are shown on **A** Tween-80 agar (*S*. *casei* CBS 206.35). **B** Skim milk agar (*Sph*. *equinum* MUCL 40625). **C** Cellulose Congo-red agar (*A*. *ruber* CBS 592.71)

Physiological tests were performed in five categories for the strains classified in *Sporendonema*. The growth rates were recorded at 4, 10, 15, 27, 30 and 36 °C. Growth was also compared using oat meal agar (OA) and OA supplemented with streptomycin and penicillin (OA/PS). Salt tolerance was evaluated using MEA containing 3, 10, 17 and 25% NaCl at 15 °C for *Sporendonema* and *Hormiscium* strains and at 24 °C for the remaining strains. Lipolytic activity was evaluated on Tween-80 agar (Ates et al. 2008), casein hydrolysis on home-made skim milk agar and cellulolytic ability on cellulose Congo-Red agar (CCA) (Gupta et al. 2012). Lipolytic, proteolytic and cellulolytic abilities were evaluated after incubation for 21 d by the formation of transparent halos and a lipolysis zone around the colonies (Fig. 1). All physiological tests were performed twice.

## Results

### Phylogeny

**Fig. 2:**
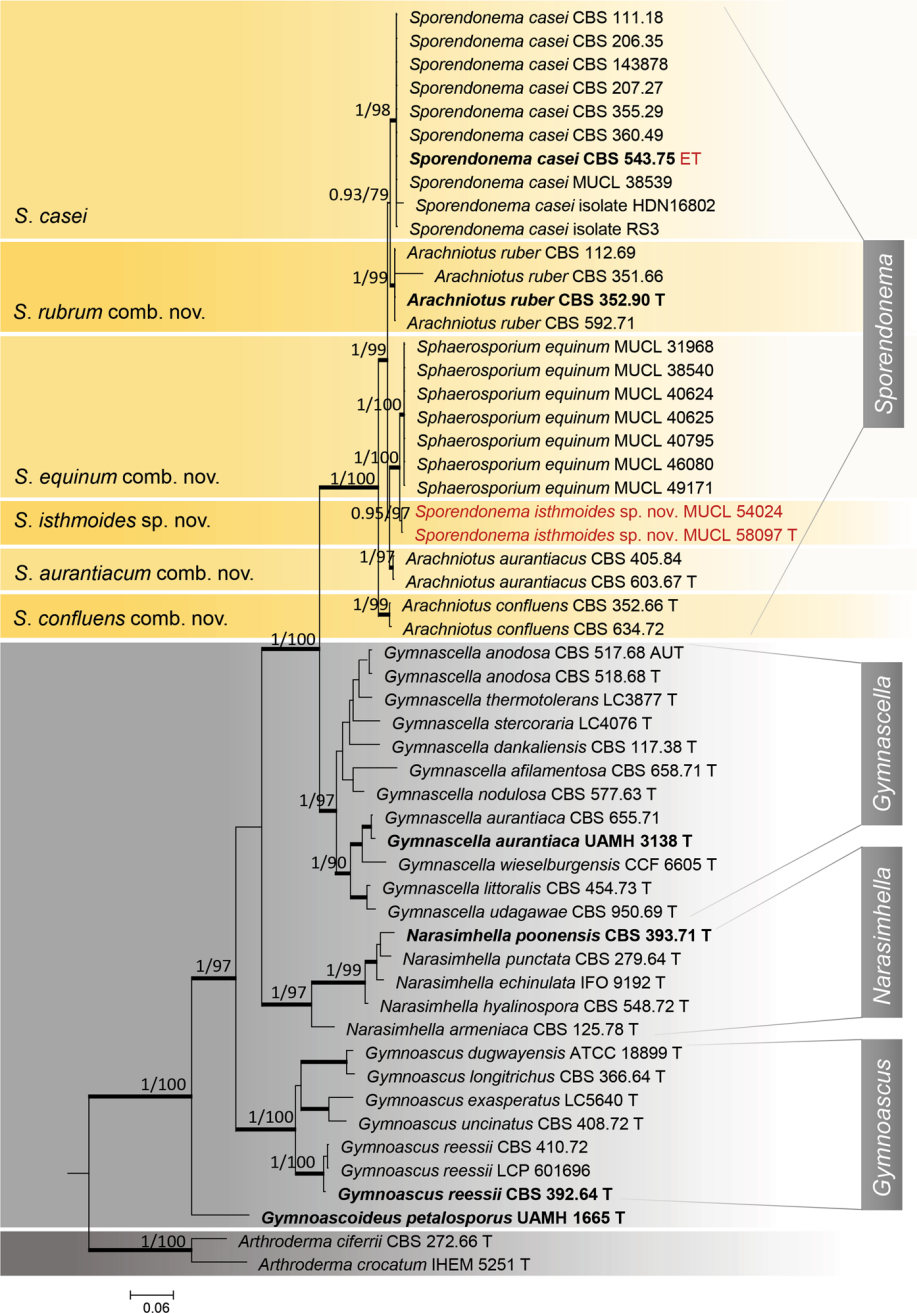
Maximum likelihood Phylogenetic tree from the combined ITS and LSU data. Type species of the genera are bold, epitype and the new species described in the present study are shown in red, newly proposed names are shown at the left side. Values of ≥ 0.95 pp for Bayesian probability and ≥ 80% for maximum likelihood are shown with thickened branches. *Arthroderma ciferrii* and *Arthroderma crocatum* were used as outgroup species

The best-fitting model was TNe + G4 for ITS and *TUB*, and TN + F + I + G4 for LSU. Compared to the analyses with ITS, LSU, and *TUB* data, ITS + LSU alone yielded better resolution among clusters (Fig. 2). In the two-marker analysis, all known supported relationships in *Gymnoascaceae*, i.e., those of *Arachniotus*, *Gymnascella*, *Narasimhella* and *Gymnoascus* designated previously, were fully confirmed by the Bayesian analysis (Fig. 2).

Species previously described in *Arachniotus* were found in different clusters based on combined data analyses of the ITS + LSU and ITS + LSU + *TUB* loci (Fig. 2; Fig. S1). *Hormiscium aurantiacum* and *S*. *casei* strains were clustered together, while another cluster was formed by *Sph*. *equinum* strains, the latter segregating into two groups compatible with their sources of isolation, i.e., cheese vs. other sources. *Arachniotus ruber* (CBS 352.90) showed 99% ITS homology with *S*. *casei* (CBS 543.75) and 98% with *Sph*. *equinum* (MUCL 46080). The ITS similarity between *S*. *casei* (CBS 543.75) and *Sph*. *equinum* (MUCL 46080) was 97%. Both *H*. *auranticaum* strains were identical to *S*. *casei* (100% of the ITS and LSU sequences; 525 bp and 803 bp, respectively).

### Physiology

All strains showed growth at 10 °C and 15 °C. *Sporendonema casei*, *H*. *aurantiacum* and two of the *A*. *ruber* strains did not grow at 27 °C and 30 °C. *Sporendonema casei* CBS 207.27, *H*. *aurantiacum* and *A*. *confluens* did not grow at 4 °C. In contrast to the remaining soil strains, strains that were isolated from agricultural soils were able to grow at 4 °C, but unable to grow at 27 °C.

All strains were able to grow in the presence of 3% and 10% NaCl. A zone of lipolysis was detected on Tween-80 agar for all strains except *A*. *ruber* CBS 351.66 and *Sph*. *equinum* MUCL 46080. Even though all strains showed growth on skim milk agar, only *A*. *aurantiacus* CBS 603.67, *A*. *confluens* CBS 352.66 and CBS 634.72, *A*. *ruber* CBS 351.66, *H*. *aurantiacum* CBS 206.35, *S*. *casei* CBS 207.27 and CBS 355.29 and *Sph*. *equinum* MUCL 40625 and MUCL 46080 were able to hydrolyse caseins. Additionally, almost all *Arachniotus* strains (7/8) were found to be able to grow on OA/PS, while the cheese isolates and strain *H*. *aurantiacum* CBS 111.18 isolated from stockfish did not grow on this medium. The results on the CCA were found to be inconsistent in repeated analyses. Only in *A*. *confluens* CBS 352.66 and *A*. *ruber* CBS 351.66 halos around their colonies on CCA remained consistently absent. The results of the physiological tests are shown in Table 2.

**Table 2:** Source information of the strains used for the physiology tests and the results after 21 days of incubation

Taxon name^1^	Accession number^2^	Source	Temperature °C						Colony growth (diameter cm)^3^								Halo zone^4^		
			4	10	15	27	30	36	PDA	OA	OA/PS	MEA	MEA3	MEA10	MEA17	MEA25	T80	SM	CCA
*S. aurantiacum*	CBS 603.67^T^	Soil	+	+	+	+	+	–	2.5	1.3	–	4.0	7.8	5.7	2.3	–	+	+	+ / +
	CBS 405.84	Dung of mouse	+	+	+	+	+	–	4.0	3.5	1.4	2.8	5.8	5.0	1.5	–	+	–	+ / +
*S*.* confluens*	CBS 352.66^T^	Dung	–	+	+	+	+	–	4.0	5.0	1.0	2.0	6.6	3.7	–	–	+	+	–/–
	CBS 634.72	Soil	–	+	+	+	+	–	4.0	3.5	1.5	3.8	5.4	4.4	1.0	–	+	+	±
*S. casei*	CBS 543.75^ET^	Cheese	+	+	+	–	–	–	0.9	1.9	–	2.1	4.6	3.8	1.2	–	+	–	+ NG
	CBS 111.18	Stockfish	–	+	+	–	–	–	1.5	2.3	–	1.5	3.9	3.8	1.0	–	+	–	±
	CBS 206.35	Cheese rind	–	+	+	–	–	–	1.5	3.0	-	2.2	4.1	2.9	1.0	–	+	+	+ / +
	CBS 207.27	Unknown	–	+	+	–	–	–	3.5	3.0	–	2.7	5.5	5.3	1.5	–	+	+	+ / +
	CBS 355.29	Unknown	+	+	+	–	–	–	0.5	2.0	–	1.0	2.7	3.1	1.4	–	+	+	+ / +
	CBS 360.49	Cheese	+	+	+	–	–	–	3.5	2.7	–	2.3	3.4	4.6	1.3	–	+	–	±
	CBS 143878	Cheese	+	+	+	–	–	–	0.5	3.0	–	1.5	4.0	4.8	1.2	–	+	–	+ / +
*S. equinum*	MUCL 40625	Cheese	+	+	+	+	–	–	1.1	1.1	–	1.1	3.6	3.6	2.3	–	+	+	NG/ +
	MUCL 46080	Sheep cheese	+	+	+	+	–	–	1.6	1.5	–	1.2	4.2	3.3	2.0	–	-	+	+ / +
*S. isthmoides*	MUCL 54024^T^	Insect pupa	+	+	+	+	–	–	6.0	5.0	2.5	3.9	5.2	4.0	2.0	–	+	–	–/ +
	MUCL 58097	Bat wing swap	+	+	+	+	–	–	4.0	3.0	0.8	2.8	5.8	4.6	2.4	–	+	–	-/ +
*S*.* rubrum*	CBS 352.90^T^	Soil	–	+	+	+	+	–	4.0	3.5	2.0	4.6	5.6	3.6	–	–	+	–	+ / +
	CBS 112.69	Wheat field soil	+	+	+	–	–	–	5.5	4.5	3.5	3.9	6.0	3.2	–	–	+	–	+ / +
	CBS 351.66	Alluvial pasture soil	+	+	+	+	+	–	6.3	5.0	1.5	7.3	7.3	1.0	–	–	–	+	–/–
	CBS 592.71	Agricultural soil	+	+	+	–	–	–	7.0	6.2	4.5	7.0	7.6	4.7	–	–	+	–	±

^1^*S* = *Sporendonema*. ^2^*CBS* Culture collection of the Westerdijk Biodiversity Institute, The Netherlands, *MUCL* Mycothèque de l’Université Catholique de Louvain, Belgium. ^*ET*^epitype strain, ^*T*^type strain. ^3^*PDA* potato dextrose agar, *OA* oat meal agar, *OA/PS* oat meal agar supplemented with penicillin and streptomycin, *MEA3* malt extract agar with 3% NaCl, *MEA10* malt extract agar with 10% NaCl, *MEA17* malt extract agar with 17% NaCl, *MEA25* malt extract agar with 25% NaCl. ^4^*T80* Tween-80 agar, *SM* skim milk agar, *CCA* Cellulose Congo-red agar, *NG* no growth

### Taxonomy

Based on the above phylogenetic and phenotypic results, the treated species previously described as *Arachniotus*, and *Sphaerosporium* are considered congeneric with *Sporendonema*, since this genus has historical nomenclatural priority. Differences in micromorphology of *Sporendonema* species are provided in Table 3.

**Table 3:** Summary of the morphological differences among *Sporendonema* species

Species	Microscopy	Spore/Hyphae shape
*S*.* aurantiacum*	Hyphae and asci can be seen on slides from MEA while slides from PDA shows chlamydospore-like structures on the hyphae. Ascospores orange-yellow, globose and smooth	
*S*.* casei*	Slides from MEA shows the typical enteroarthric conidiogenesis while spherical orange spores and irregular hyphae can be seen only on the slides from PDA	
*S*. *confluens*	Ascospores are yellow but compared to *S*. *rubrum* and *S*. *aurantiacum* they have lighter color. Ascospores have slightly thick walls. Racquet hyphae are common on slides from MEA	
*S*. *equinum*	Holoblastic conidiogenesis with smooth and spherical conidia. Longer conidia chains compared to *S*. *isthmoides*	
*S*. *isthmoides*	Thallic-enteroarthric conidiogenesis with mostly lemon-shaped and warted conidia. Reveals remnants of hyphae between the conidia. Hyphae with warts are also common	
*S*. *rubrum*	Asci can be seen on slides from MEA while rocket hyphae are common on slides from PDA. Ascospores are orange-yellow, oblate with two equatorial thickenings	

***Sporendonema*** Desm. – Annls Sci. Nat., Sér. 1 11: 246 (1827).

** = ***Coprotrichum* Bonord.—Handb. Allgem. Mykol. (Stuttgart): 76 (1851).

 = *Arachniotus* Schröt.—Krypt.-Fl. Schlesien (Breslau) 3.2(1–2): 210 (1893) [1908].

Type species: *Sporendonema casei* Desm.

***Sporendonema aurantiacum*** (Kamyschko) Kandemir & de Hoog, **comb. nov.**

Figure 3A–D, M, N

**Fig. 3:**
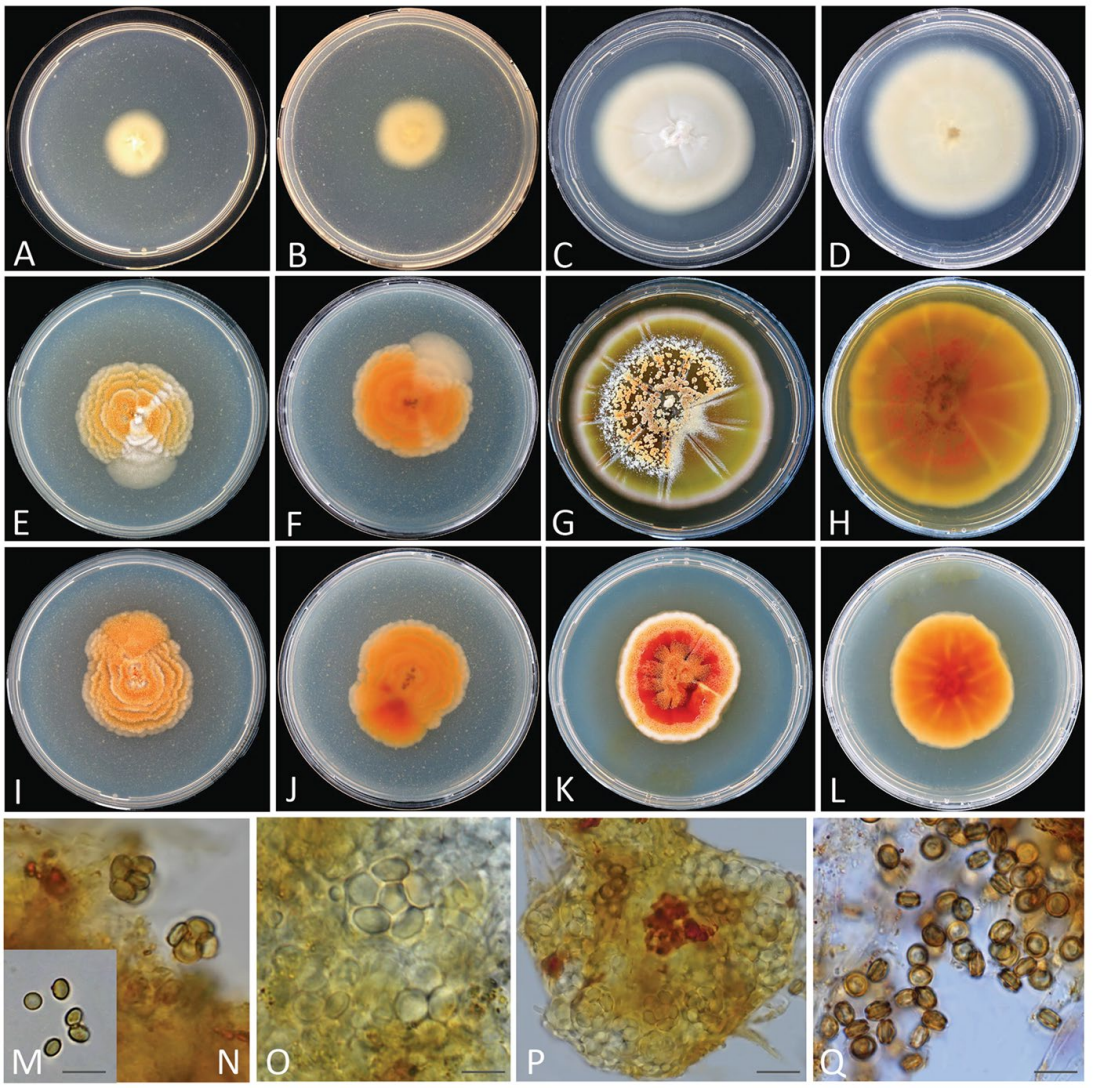
**A‒D, M, N** *Sporendonema aurantiacum* CBS 603.67. **E‒H, O, P** *Sporendonema confluens* CBS 352.66. **I‒L, Q** *Sporendonema rubrum* CBS 352.90. **A, B, E, F, I, J** Colony surface and reverse on PDA after 21 d at 24 °C. **C, D, G, H, K, L** Colony surface and reverse on YpSs agar after 21 d at 24 °C. **M‒Q** Asci and ascospores. Scale bars **M‒Q** = 10 μm

MycoBank number: MB842801.

≡ *Pseudoarachniotus aurantiacus* Kamyschko – Nov. Sist. Niz. Rast. 4: 224 (1967) ≡ *Arachniotus aurantiacus* (Kamyschko) v. Arx - Persoonia 6(3): 373 (1971).

*Holotype* Russia, Republic of Kalmykia, from semi-desert (slightly loam) soil, Culture 4–1/2, (Kamyschko 1967), was preserved Institute of Antibiotics, Saint-Petersburg (Leningrad). *Ex-holotype culture* CBS 603.67. *Alternative collection numbers* BKM F-1140, ATCC 22394, NRRL A-18287, BKM F-1140, and UAMH 3529.

*Notes*: *Sporendonema aurantiacum* has globose, and smooth ascospores without a prominent equatorial rim similar to that of *S*. *confluens*. *Sporendonema aurantiacum* can be differentiated from *S*. *confluens* by its darker ascospores that has discoid from the side view. A detailed description has been provided by von Arx (1970).

***Sporendonema casei*** Desm. – Annls Sci. Nat., Sér. 1, 11: 246 (1827).

Figure 4A–M

**Fig. 4:**
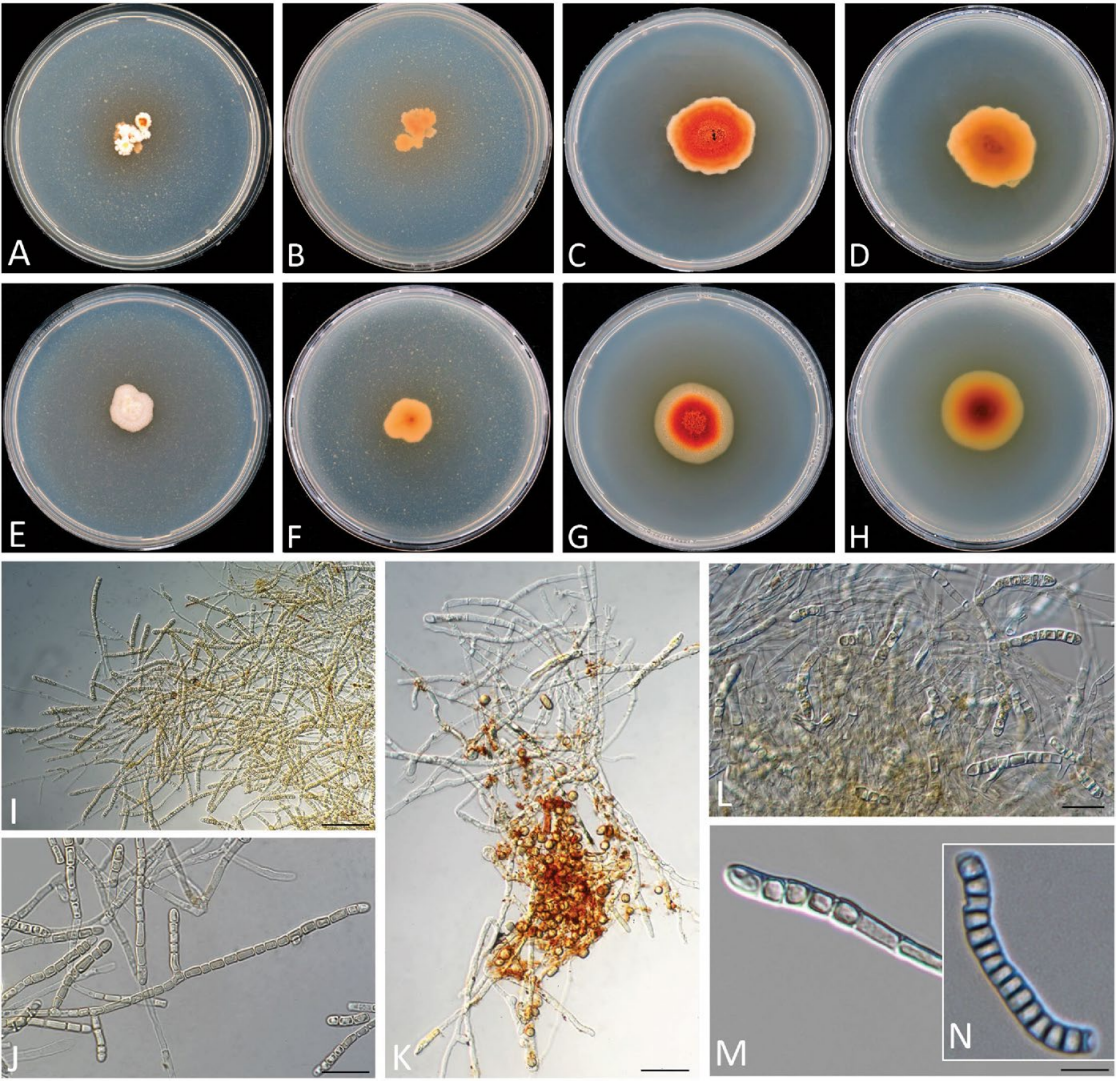
**A‒M** *Sporendonema casei* **A, B, I‒K** CBS 543.75, **E‒H, L, M** CBS 206.35**. N** *Hormiscium aurantiacum* type specimen ILLS 36355. **A, B, E, F** Colony surface and reverse on PDA after 21 d at 15 °C. **C, D, G, H** Colony surface and reverse on YpSs agar after 21 d at 15 °C. **I, J** Mass of thallic-arthric conidiophores, slides from MEA. **K** Mass of hyphae, conidiophore, and conidia, slide from PDA. **L** Hyphae and conidiophores. **M, N** Conidiophore developing conidia. Scale bars **I‒N** = 10 μm

  =  *Torula sporendonema* Berk. & Broome – Ann. Mag. Nat. Hist., Ser. 2, 5: 460 (1850).

*Holotype* material is not known to be preserved. *Lectotype* (designated here, MBT 10017580), drawings in Desmazières (1827) plate 21A, Fig. 1. *Epitype* (designated here, MBT 10017581) CBS 543.75, isolated from cheese, by Sochal, 1975, preserved in metabolically inactive state.

*Notes*: *Sporendonema casei* is a well-known cheese-inhabiting fungus that produces orange-red spots on cheese. This slow-growing and xerotolerant fungus produces cubical conidia with rounded corners from club-shaped hyphae by enteroarthric conidiogenesis. A detailed description of *S*. *casei* has been provided by Sigler and Carmichael (1976).

***Sporendonema confluens*** (Sartory & Bainier) Kandemir & de Hoog, **comb. nov.**

Figure 3E–H, O, P

MycoBank number: MB842802.

≡ *Gymnoascus confluens* Sartory & Bainier – Bull. Soc. Mycol. Fr. 29: 261 (1913) ≡ *Arachniotus confluens* (Sartory & Bainier) Apinis – Mycol. Pap. 96: 37 (1964) ≡ *Gymnascella confluens* (Sartory & Bainier) Currah – Mycotaxon 24: 75 (1985).

*Neotype* UK, London, Birbeck College, from dung, 1959, dry culture BDUN 375, designated by Apinis (1964). *Alternative collection numbers* ATCC 22220, CBS 352.66, IMI 100873, NRRL 5979, Orr O-3559, and UAMH 3565.

*Notes*: See the notes under the *S*. *auranticum* section. Detailed description has been provided by Currah (1985) and Apinis (1964)

***Sporendonema equinum*** (Desm.) Kandemir, Decock & de Hoog, **comb. nov.**

Figure 5

**Fig. 5:**
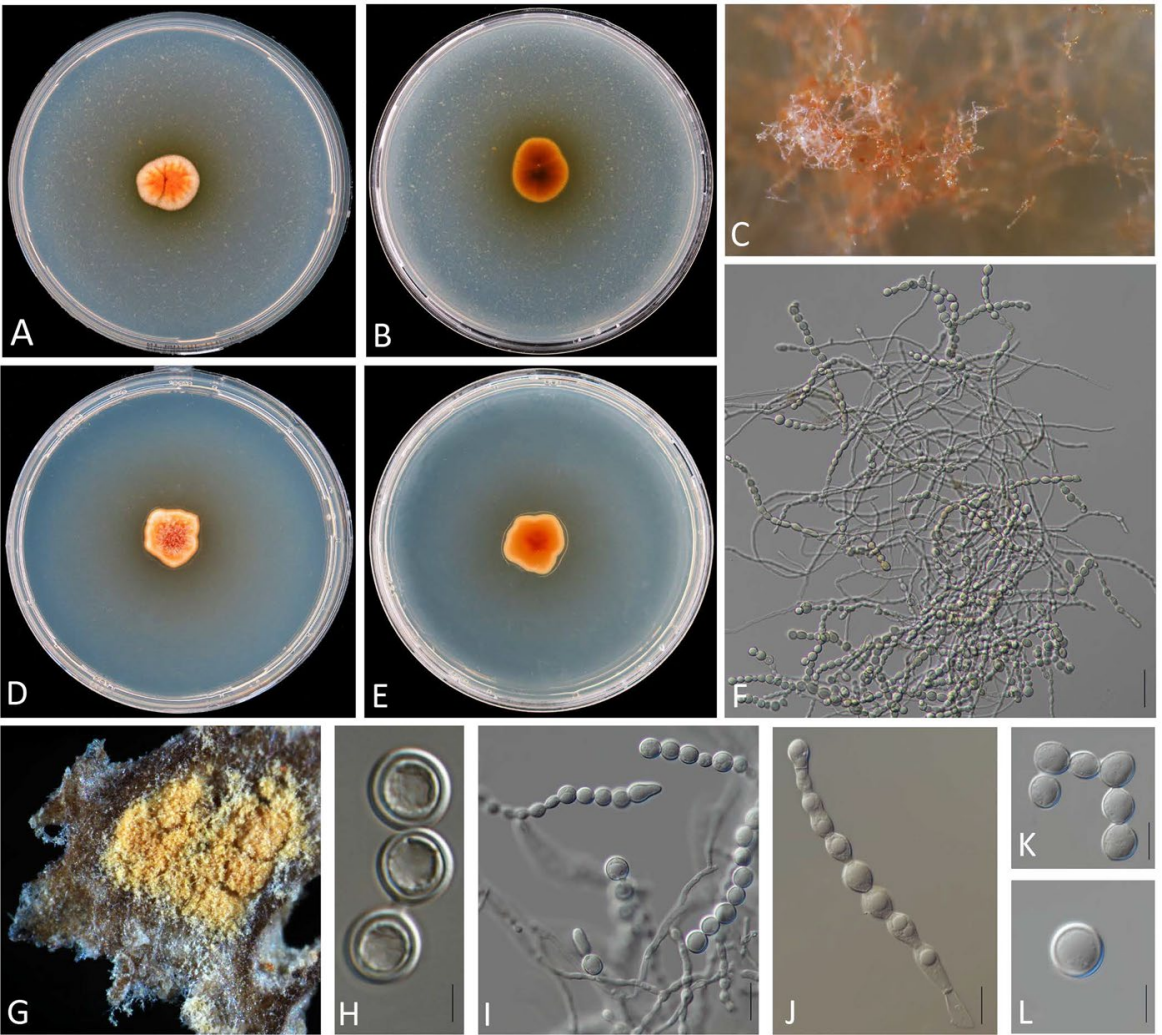
**A‒L** *Sporendonema equinum* **A‒F**, **I‒L** MUCL 46080, **G, H** type specimen ILLS 45141. **A, B** Colony surface and reverse on PDA after 21 d at 24 °C. **C** Colony details on YpSs agar 21 d at 24 °C. **D, E** Colony surface and reverse on PDA after 21 d at 24 °C. **F** Spore chains, conidiophores and hyphae. **G** Colonies on horse hooves. **H** Thick-walled spores obtained from the type specimen. **I** Catenate conidia. **J** Conidiophores and conidia. **K, L** Conidia. Scale bars **F, H‒L** = 10 μm

MycoBank number: MB842804.

≡ *Torula equina* Desm. – Annls Sci. Nat., Bot., Sér. 4, 4: 126 (1855) ≡ *Oospora equina* (Desm.) Sacc. & Voglino – Syll. Fung. (Abellini) 4: 22 (1886) ≡ *Sphaerosporium equinum* (Desm.) Crane & Schokn. – Mycologia 78(1): 86 (1986).

*Isoneotype* France, from old and humid horse hoofs, collected and identified by Desmazieres, ILLS 45141, collector number H. G. 1510, designated by Crane and Schoknecht (1986).

*Notes*: *Sphaerosporium* was introduced by von Schweinitz (1834) based on morphology of the type species *Sph*. *lignatile* found growing on dead wood in the USA. The holotype for *Sph*. *lignatile* was designated as #3036 (PH, Paris Herbarium). Later, *Sph*. e*quinum*, originally described as *Torula equina*, was added to the genus (Crane and Schoknecht 1986). Partridge and Morgan-Jones (2002) reviewed *Sphaerosporium* and provided descriptions for both *Sph*. *lignatile* and *Sph*. *equinum*. Authors noted that despite their substrate differences, these two taxa share morphological similarities suggesting a close relationship (Partridge and Morgan-Jones 2002).

However, molecular analyses do not support any relationship between *Sph*. *lignatile* and *S*. *equinum* (Song et al. 2019). In the current study, micromorphology of the type specimen of *Torula equina* ILLS 45141 was examined. The conidia were abundant, arranged in basipetal chains, globose, with thick, and smooth walls (Fig. 5).

Additionally, we examined MUCL 46080, which was isolated from the rind of a sheep cheese in France, as a reference strain to evaluate morphological characteristics and physiology of *Sph*. *equinum*. Since we were not able to obtain a pure culture from the type specimen, we could not compare the type and the cheese isolates phylogenetically. Nevertheless, we propose a new combination for the cheese isolates of the *Sph*. *equinum* since they are classified within *Sporendonema*, *Onygenales*, *Eurotiomycetidae* (Kandemir et al. 2022), while *Sph*. *lignatile* is classified in *Pezizales*, *Pezizomycetidae* (Song et al. 2019).

The strains UAMH 11516 (= MUCL 58097), obtained from the skin of bat wings, and MUCL 54024, from insect pupa were found to be phylogenetically related to the cheese isolates of *Sph*. *equinum* (Fig. 2). However, the growth rate on PDA, OA, OA/PS and MEA, the conidial shape and size, and caseinase activity were different between the two groups. Therefore, a new species was described to accommodate MUCL 54024 and MUCL 58097.

***Sporendonema isthmoides*** Decock, Kandemir, Hern.-Rest. & de Hoog, **sp. nov.**

Figure 6

**Fig. 6:**
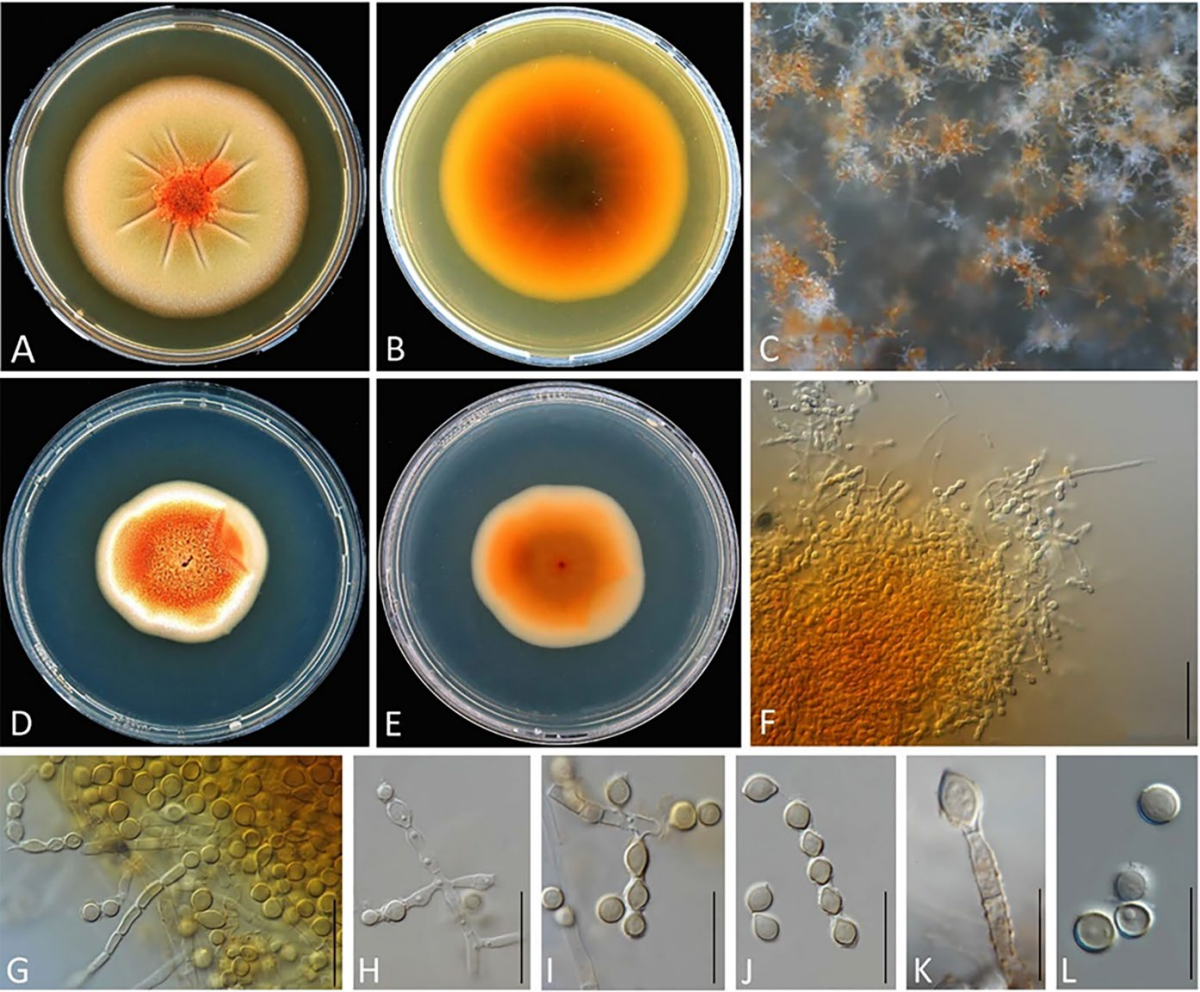
**A‒L** *Sporendonema isthmoides* MUCL 58097. **A, B** Colony surface and reverse on PDA after 21 d at 24 °C. **C** Colony details on OA/PS. **D, E** Colony surface and reverse on YpSs agar after 21 d at 24 °C. **F** Mass of hyphae and conidia. **G** Arthric conidiophore. **H, I** Enteroarthric conidiophore. **J** Hyphal remnants on catenate conidia. **K** Fertile hyphae with warts. **L** Thick-walled, single-cell conidia. Scale bars **F‒L** = 50 μm

MycoBank number: MB842809.

*Etymology* In Greek “isthmus” means “neck”, and “isthmoides” is used for “resembling isthmus”, referring to the narrow conjunction between conidia in chains.

*Holotype* Canada, New Brunswick, Berryton Cave, from swab sample of living female little brown bat (*Myotis lucifugus*) skin, 2010, isolated by K. J. Vanderwolf, dried culture UAMH 11516, preserved in a metabolically inactive state. *Alternative collection number* MUCL 58097; *GenBank numbers* ITS: OM468607, LSU: OM515118, *TUB*: OM616026. *Additional specimen* Belgium, insect pupa in the attic of a house, 2012, C. Decock, MUCL 54024; *Genbank numbers* ITS: OK255531, LSU: OK255535.

*Vegetative hyphae* hyaline, septate, smooth, 2.5–4.5 µm wide; *fertile hyphae* mostly smooth and some ornamented with warts (Fig. 6); conidiogenesis thallic-enteroarthric; *conidia* hyaline to pale yellow, yellow-orange in mass, 1-celled, lemon-shaped in chains and becoming globose when separated, truncated at one or both ends, smooth- and thick-walled, occasionally with warts; 6–8.5 × 3.5–5 µm. *Sexual morph* not observed.

*Culture characteristics* on PDA reaching 40 mm diam after 21 d at 24 °C; flat, elevated in the center; margin regular; obverse color orange, dirty white-beige at the periphery (Fig. 6A); reverse dark brown at the center and orange-yellow at the periphery (Fig. 6B). Colonies on YpSs agar reaching 38 mm diameter after 21 d at 24 °C, flat, slightly elevated at the margin, texture velvety, obverse color orange with cream-white edges (Fig. 6D); reverse orange with a cream-colored periphery (Fig. 6E).

*Growth temperatures* minimum 4 °C and maximum 27 °C.

*Physiology* Casein not hydrolysed. Growth present at NaCl concentrations of 3, 10 and 17 but not 25% (w/w).

**Fig. 7:**
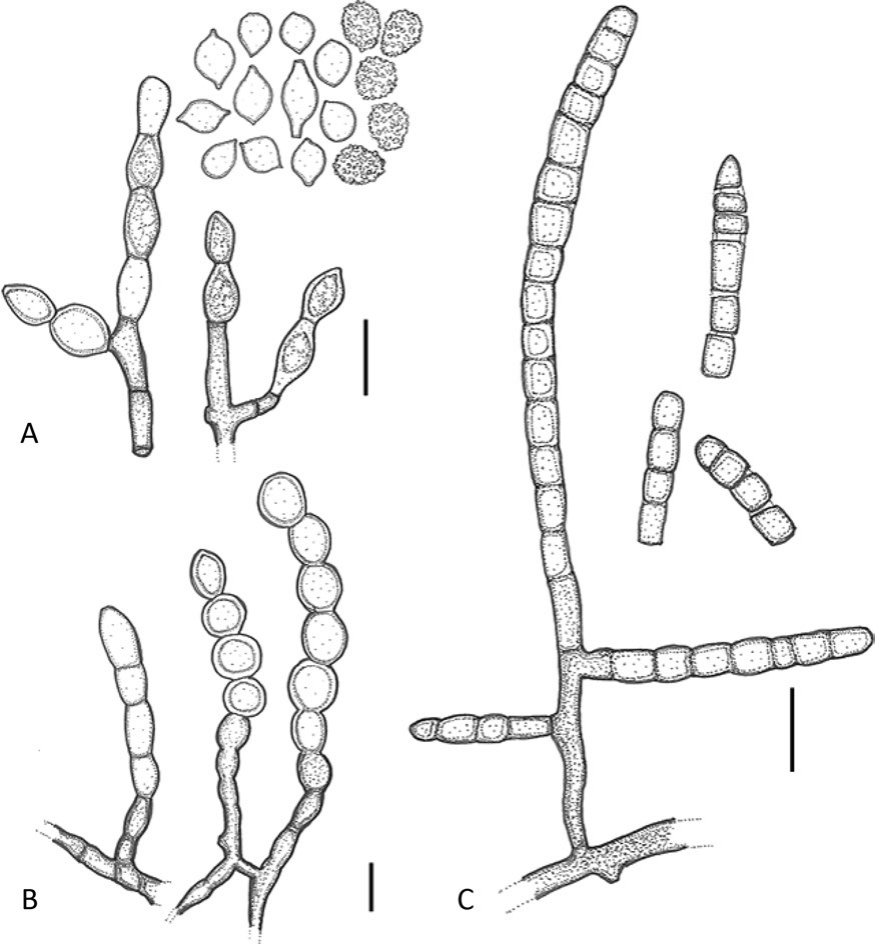
Line drawing of conidiophores developing conidia. **A** *Sporendonema isthmoides*. **B** *S*. *equinum*. **C** *S*. *casei*. Bar = 10 μm

*Notes*: Based on ITS and LSU data analyses, the phylogenetically closest species to *S*. *isthmoides* is *S*. *equinum*. *Sporendonema isthmoides* and *S*. *equinum* differ morphologically in conidiogenesis (thallic-enteroarthric vs holoblastic; Figs. 5 and 6), shape of conidia in chains (lemon-shaped vs. globose) and size (9.5 × 13 µm vs. 4.0 × 7.5 µm). The conidia and hyphae of *S*. *equinum* are smooth-walled, whereas some conidia and hyphae of *S*. *isthmoides* are warted. *Sporendonema isthmoides* grows faster than *S*. *equinum* on almost all tested media (PDA, OA, and MEA supplemented with 3% and 10% NaCl at 24 °C; Table 2), can grow on OA/PS medium and lacks caseinase activity. Differences in the micromorphology of *S*. *casei*, *S*. *equinum* and *S*. *isthmoides* are illustrated in Fig. 7.

***Sporendonema rubrum*** (Tiegh.) Kandemir & de Hoog, **comb. nov.**

Figure 3I–L, Q

MycoBank number: MB842803.

≡ *Gymnoascus ruber* Tiegh. – Bull. Soc. Bot. Fr. 24: 159 (1877) ≡ *Arachniotus ruber* (Tiegh.) Schröt. – Krypt. -Fl. Schlesien (Breslau) 3.2(1–2): 210 (1893) [1908].

*Neotype* UK, from soil, IMI 92796, designated by Kuehn and Orr (1964). *Alternative collection numbers* CBS 352.90 and ATCC 15315.

*Notes*: *Arachniotus ruber* was described from coyote dung as type species of the genus *Arachniotus* and was outstanding with its low temperature (5 °C) requirement for isolation (Currah 1985). It has hyaline asci, orange-yellow and smooth ascospores with two equatorial lines (Fig. 3Q). A detailed description of the fungus is given by Kuehn and Orr (1964).

## Discussion

Nomenclaturally, *Sporendonema casei* is the oldest described species in the family *Gymnoascaceae*. It was introduced by Desmazières (1827) for an orange-red fungus growing on cheese. After several disagreements on the nomenclature and the taxonomic position of this “red mould” (Corda 1838; Berkeley and Broome 1850; Saccardo 1882; Bainier 1907), the name *S*. *casei* became widely accepted (Hammer and Gilman 1944; von Arx 1970). Ropars et al. (2012) and Kandemir et al. (2022) confirmed its placement in the *Gymnoascaceae*, *Onygenales*. No type specimen of *S*. *casei* is known to be preserved, and although the species was included in several subsequent studies (Hammer and Gilman 1944; Sigler and Carmichael 1976; Ropars et al. 2012), no type culture has been indicated. To stabilize the nomenclature, we therefore proposed the strain CBS 543.75, isolated from cheese, as epitype.

*Sphaerosporium equinum* was originally described from a keratinous source (Desmazières 1855, Fig. 5G). However, all strains that were subsequently analysed under this name were isolated from cheese. This could be a result of a lack of sampling from different keratinous substrates. Such that, horse hooves contain beta (β) keratin similar to that of reptiles and birds which is different from that of other mammals containing alpha (α) keratin as the major component (Greenwold et al. 2014; Kakkar et al. 2014). Possibly cheese isolates had been misidentified in the past.

Two strains with superficial similarity to *Sph*. *equinum* were derived from other sources than cheese: UAMH 11516 (= MUCL 58097) isolated from a bat wing and MUCL 54024 from an insect pupa. These two strains were also phylogenetically different from the cheese isolates (Fig. 2). In addition, their growth rate, caseinase activity, and ascospore size were also found to be different. Therefore, these two strains were introduced here as a new species. In bat wings, sensory hairs were made of α-keratin (Khan et al. 2014). Insect pupa cocoon structure contains silk which has a different form of β-keratin (Palmer and Bonner 2011). It was also reported that insects contain high quantity of fatty acids in their pupal life stage (Meetali et al. 2014; Smets et al. 2020) which might be a source of nutrition for the fungi grown on this substrate.

Two strains identified as *H*. *aurantiacum* were preserved in the CBS collection: CBS 111.18 and CBS 206.35, both originating from salted environments, i.e., cheese and stockfish. These strains produced red–orange colonies (Fig. 4E–H) similar to those of the *S*. *casei* strains in the present study. In addition to colony morphology, these two *H*. *aurantiacum* strains share the similar habitat and micromorphology as well as the identical ITS, LSU, and *TUB* sequences with *S*. *casei*. Therefore, the strains CBS 111.18 and CBS 206.35 were regarded as previously misidentified and corrected here as *S*. *casei*.

Soil, dung, and, fluvial sediments are common sources for onygenalean fungi, and xerophilic and halophilic capacities are characteristic for certain families, such as *Ascosphaeraceae* and *Spiromastigoidaceae* (Kandemir et al. 2022, Torres-Garcia et al. 2023). In contrast, species of *Sporendonema*, *Sphaerosporium* and *Arachniotus* are classified in *Gymnoascaceae*, and are able to grow on substrates with low water activity, such as cheese, dried meat products, and desert soil (Ropars et al. 2012; Scaramuzza et al. 2015). The cheese rind, the prevalent source of isolation of *Sporendonema*, has high free fatty acid, protein, and salt contents (Kandemir et al. 2022), and in line with this, all cheese isolates were able to tolerate 17% NaCl and showed lipolytic activity in the present study. The only strain lacking lipolysis, MUCL 46080, was isolated from sheep cheese, which forms a soft and bloomy rind different from those of hard cheeses.

Morphologically, *S*. *casei* strains yielded hyphae that produce enteroarthric conidia with thick walls and rounded corners (Fig. 4). *Spharosporium equinum* showed holoblastic conidia with thick walls and was mostly smooth and oblate; *S*. *isthmoides* yielded thick-walled, oblate, lenticular conidia produced by thallic-enteroarthric conidiogenesis and showing a distinct point of attachment (Fig. 6). Ascus formation together with arthroaleuriospore production was observed only for *A*. *aurantiacum, A*. *confluens,* and *A*. *ruber* (Fig. 3). Nevertheless, these morphological variations did not interfere with the phylogenetic classification of the species in a single genus.

In general, multilocus sequencing data are applied to delimitate fungal species (Giraldo et al. 2014; Kandemir et al. 2020; Crous et al. 2021; Geiser et al. 2021; Hainsworth et al. 2021). In the current dataset, the combined data of ITS + LSU + *TUB* did not reveal significant differences from those obtained with only ITS + LSU data. Similarly, Ropars et al. (2012) also did not find a major difference between the phylogenetic trees constructed with *TEF*1 + *TUB* loci and ITS + LSU sequences of *Arachniotus*, *Sporendonema* and *Sphaerosporium* strains. As all genes yield a similar, stable phylogenetic topology, ITS alone is sufficient to identify *Sporendonema* species (Fig. S2).

## Conclusion

Based on phylogenetic data, species previously described as *Arachniotus aurantiacus*, *A*. *confluens, A*. *ruber*, *Sphaerosporium equinum* and *Sporendonema casei* are congeneric. These fungi represent halophilic, psychrophilic, and xerotolerant members of the *Gymnoascaceae*. Differences in conidial morphology, cellulolytic and lipolytic ability, casein degradation and maximum temperature of growth are variable between species and even among strains of the same species, but insufficient for accommodating these species in different genera. The individual species within the genus can be recognized by rDNA ITS as a primary barcode.

## Supplementary Information

Below is the link to the electronic supplementary material.

Supplementary file1 (PDF 623 KB)

## Data Availability

All data generated or analysed during this study are included in this published article and its supplementary information files. Sequence alignments and the phylogenetic trees are available in the TreeBASE (TB2:S29250) and figshare repositories (10.6084/m9.figshare.23284661).

## Declarations

### Human and animal participants

This article does not contain any studies with human participants or animals performed by any of the authors.
