# Supplementary material for: 200 years of taxonomic confusion: Sporendonema and allies
Source: Antonie Van Leeuwenhoek. 2024 Mar 14;117(1):53. doi: 10.1007/s10482-024-01935-3 (PMC10940481; doi:10.1007/s10482-024-01935-3)
Supplement: Supplementary file 1 — Supplementary file1 (PDF 623 KB) [file 10482_2024_1935_MOESM1_ESM.pdf]

Supplementary Materials

200 years of taxonomic confusion: *Sporendonema* and allies

Hazal Kandemir[1,2], Cony Decock[3], Margarita Hernández-Restrepo[2], Roman Labuda[4,5], Jos Houbraken[2], Macit Ilkit[6], G. Sybren de Hoog[1*]

[1] Center of Expertise in Mycology, Radboud University Medical Center/Canisius Wilhelmina Hospital, Nijmegen, The Netherlands.

[2] Westerdijk Fungal Biodiversity Institute, Utrecht, The Netherlands.

[3] Mycothéque de l'Université Catholique de Louvain, Louvain-la-Neuve, Belgium.

[4] Research Platform Bioactive Microbial Metabolites, Tulln/Donau, Austria.

[5] Institute of Food Safety, Food Technology and Veterinary Public Health, University of Veterinary Medicine Vienna, Veterinaerplatz 1, 1210, Vienna, Austria.

[6] Division of Medical Mycology, Faculty of Medicine, University of Çukurova, Adana, Turkey

***Author for correspondence:**

G. Sybren de Hoog

Email: Sybren.deHoog@radboudumc.nl

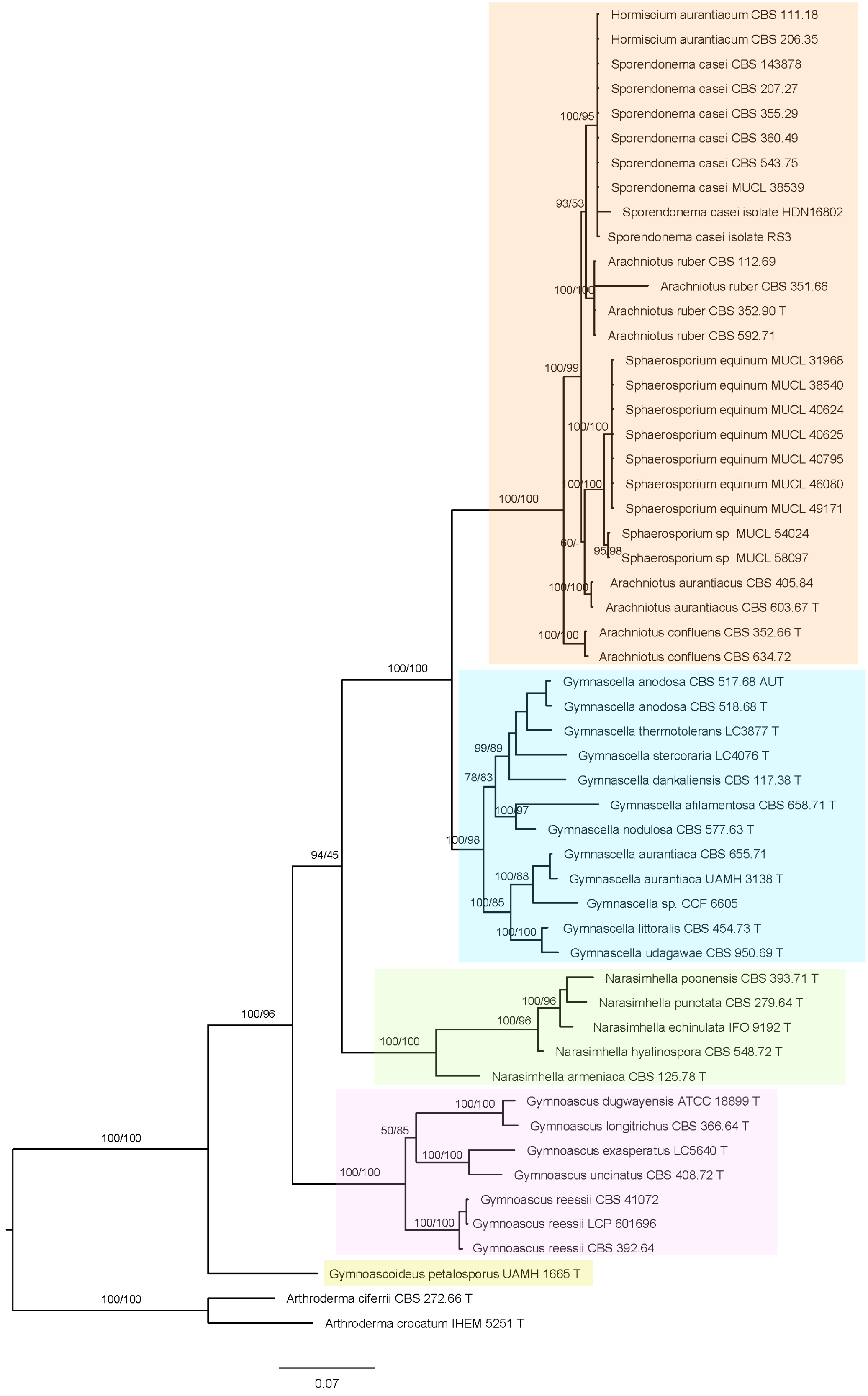

Figure S1 Multilocus (ITS, LSU and *TUB*) phylogeny of the species based on Bayesian and maximum likelihood analysis.

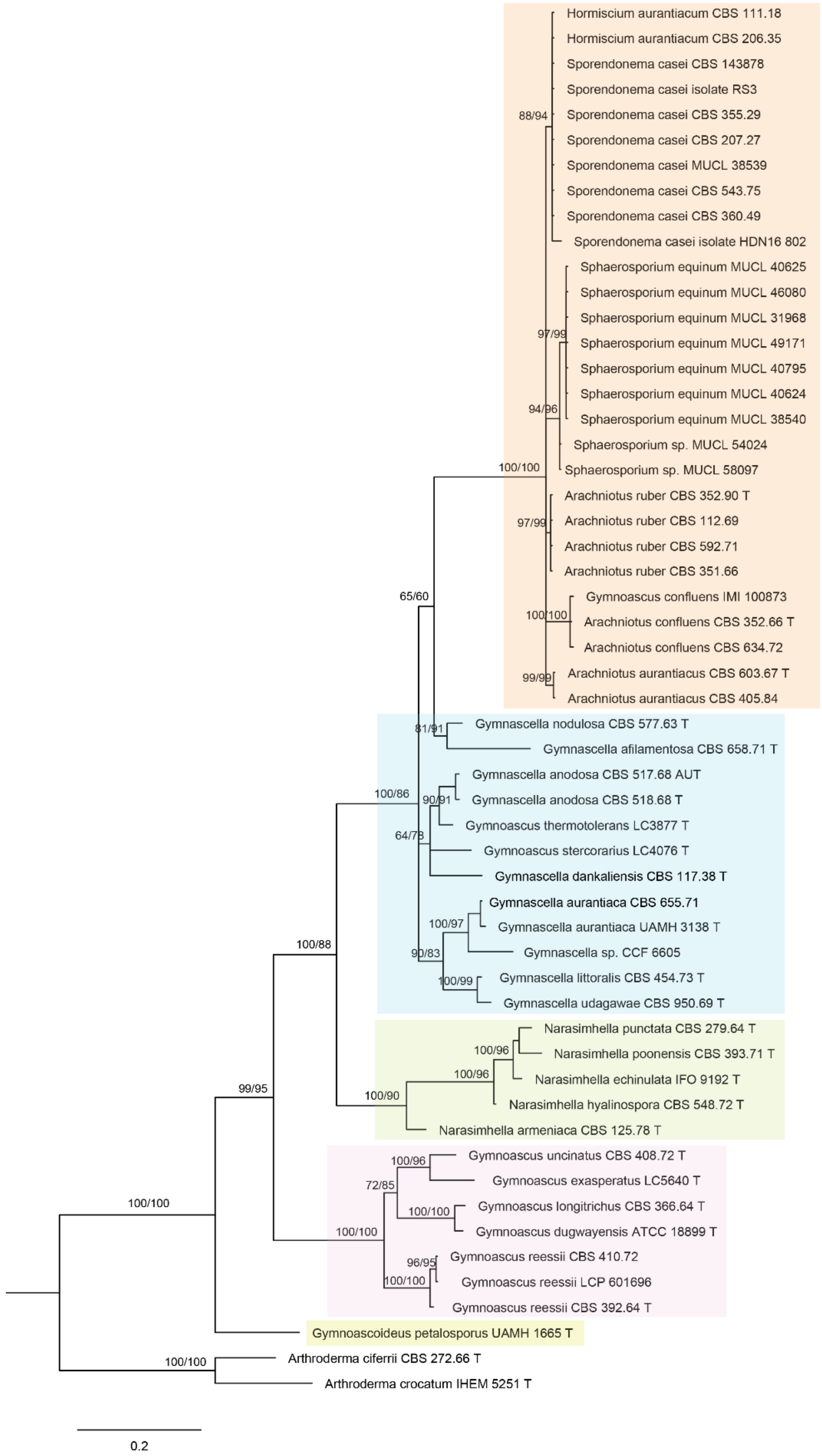

Figure S2 ITS phylogeny of the species based on Bayesian and maximum likelihood analysis
